# Clinical Trajectories of Neurodegenerative Diseases in Older Adults: A Three-Sphere Framework for Precision Geriatric Neurology

**DOI:** 10.3390/life16050827

**Published:** 2026-05-16

**Authors:** Crescenzo Testa, Francesco Palmese, Stefano Boni, Marco Domenicali, Fulvio Lauretani

**Affiliations:** 1 Department of Medicine and Surgery, University of Parma, 43126 Parma, Italy; crescenzo.testa@unipr.it; 2 Department of Medical and Surgical Sciences, Alma Mater Studiorum University of Bologna, Ravenna Campus, 48123 Ravenna, Italy; francesco.palmese2@unibo.it (F.P.); m.domenicali@unibo.it (M.D.); 3 Internal Medicine Unit Addressed to Frailty and Aging, Department of Primary Health Care, “S. Maria Delle Croci” Hospital, AUSL Romagna, 48121 Ravenna, Italy; 4 Geriatric Unit, AUSL Romagna, 48018 Faenza, Italy; stefano.boni@auslromagna.it

**Keywords:** neurodegenerative diseases, dementia, Alzheimer’s disease, Parkinson’s disease, dementia with Lewy bodies, frontotemporal dementia, clinical trajectories, polypharmacy, delirium, geriatric neurology, multimorbidity, deprescribing

## Abstract

Neurodegenerative diseases are among the most consequential disorders of later life, not only because of their increasing prevalence, rising from approximately 1–2% at age 65 to over 30% by age 85, but also because they develop within the broader clinical context of ageing, multimorbidity, frailty, and polypharmacy. In older adults, these conditions rarely present as isolated and static diagnostic entities; rather, they unfold as dynamic clinical trajectories involving the progressive interaction of cognitive decline, behavioural-neuropsychiatric symptoms, and extrapyramidal-motor dysfunction. In this review, we propose a trajectory-based framework for the interpretation and management of major neurodegenerative disorders in later life, including Alzheimer’s disease, frontotemporal dementia, Parkinson’s disease and Parkinson’s disease dementia, dementia with Lewy bodies, and vascular cognitive impairment. Building on a conceptual model organized around three major symptom spheres: cognitive, behavioural-neuropsychiatric, and extrapyramidal-motor, we argue that each disorder can be understood according to the relative predominance and temporal evolution of these domains. Alzheimer’s disease is typically cognition-led, frontotemporal dementia behaviour-led, and Parkinsonian syndromes motor-led, whereas dementia with Lewy bodies shows early multidomain convergence across all three spheres simultaneously. Vascular and mixed dementias follow more heterogeneous trajectories shaped by lesion burden, network disruption, and copathology. This framework has direct implications for diagnosis, prognostic stratification, and treatment selection, because interventions targeting one sphere may destabilize another and generate prescription cascades, delirium, or functional decline. We further discuss how biomarker-based diagnosis, disease-modifying therapies, non-pharmacological interventions, multidisciplinary care, deprescribing strategies, and palliative planning can be integrated within a trajectory-based approach. Interpreting neurodegeneration through clinical trajectories rather than diagnostic labels alone offers a more realistic and therapeutically useful model for precision geriatric neurology across the full course of disease.

## 1. Introduction

**Figure 1:**
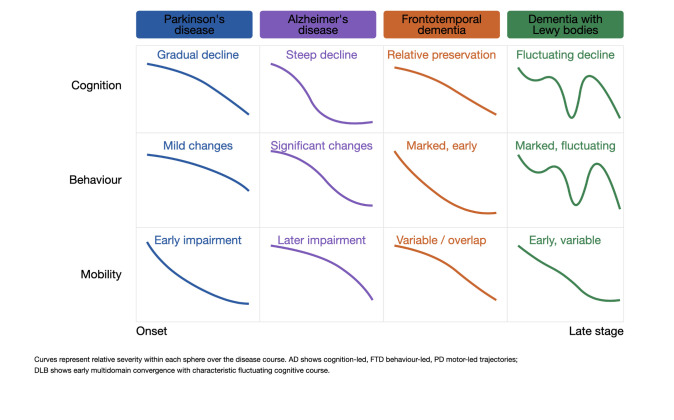
Comparative clinical trajectories of major neurodegenerative diseases across three symptom spheres: cognition, behaviour, and mobility. Parkinson’s disease shows early mobility impairment with gradual cognitive decline; Alzheimer’s disease shows steep cognitive decline with later mobility involvement; frontotemporal dementia shows marked behavioural changes with relative cognitive preservation; dementia with Lewy bodies shows early simultaneous involvement of all three spheres with a fluctuating cognitive course.

Population ageing has profoundly changed the clinical landscape of medicine. Over the last decades, the burden of disease has shifted from acute, often infectious conditions toward chronic non-communicable disorders, many of which now dominate late-life morbidity, disability, and mortality. Seven of the 10 leading causes of mortality worldwide are now chronic diseases, and among these, neurodegenerative conditions occupy a particularly prominent and growing position [1,2]. The global prevalence of dementia reached over 57 million people in 2021, with projections indicating this number will triple by 2050. Age-specific data reveal the steep relationship between neurodegeneration and ageing: dementia prevalence increases from 1–2% at age 65 to approximately 30% by age 85, and Alzheimer’s disease incidence rises from 3.4 new cases per 1000 person-years at ages 65–74 to 36 new cases per 1000 person-years at age 85 and older. Beyond dementia, Parkinson’s disease, the second most common neurodegenerative disorder, follows a similarly steep age-related prevalence curve. Collectively, neuropsychiatric conditions including dementias have contributed unfavourably to non-communicable disease mortality trends in most high-income countries since 2010 [1,2]. However, neurodegenerative disorders in older adults are rarely encountered as pure clinicopathological entities. They arise in patients who often already live with cardiovascular disease, diabetes, sleep disorders, depression, sensory deficits, sarcopenia, osteoarthritis, chronic pain, and extensive drug exposure. Approximately 46–48% of older adults globally have two or more chronic conditions simultaneously, with the proportion exceeding 83% among adults aged 85 and older. Polypharmacy, affecting approximately 15–23% of older adults, accompanies multimorbidity and creates substantial risk for drug–drug interactions, adverse effects, and prescription cascades, wherein medications introduced to control one symptom provoke adverse effects misinterpreted as new disease manifestations, leading to further prescriptions and progressive clinical destabilization [3,4]. Beyond their clinical heterogeneity, the major neurodegenerative disorders are unified by partially overlapping molecular mechanisms, including protein misfolding and aggregation, neuroinflammation, oxidative stress, mitochondrial dysfunction, synaptic failure, and progressive disruption of large-scale neural networks. These shared biological substrates help explain why clinical domains, cognition, behaviour, and motor function do not evolve in isolation but intersect and interact as disease progresses [1,2,4]. Prior conceptual approaches to neurodegenerative disease in older adults have largely operated within categorical diagnostic frameworks, organized around consensus criteria such as the DSM-5 and ICD-11, biomarker-based staging models such as the Alzheimer’s Association ATN framework, or severity-based instruments such as the Clinical Dementia Rating (CDR) and the Functional Assessment Staging Test (FAST). While these frameworks have been fundamental in standardizing diagnosis and grading disease severity, they were not designed to guide the management of cross-domain therapeutic interactions, anticipate iatrogenic risk across functional systems, or support individualized care decisions in the context of multimorbidity and polypharmacy that characterize real-world geriatric practice. The trajectory-based perspective proposed in this review does not replace these frameworks; rather, it complements them by adding a temporal and domain-interactive dimension that is particularly relevant when the clinical question is not only “what disease does this patient have” but “how is it moving across cognition, behaviour, and motor function, and what are the treatment implications of that movement”. The present review is built around a central conceptual proposition: neurodegenerative diseases should be understood not only as diagnostic categories, but also as clinical trajectories. This perspective is organized around three principal symptom spheres: cognitive, behavioural-neuropsychiatric, and extrapyramidal-motor, and proposes that different diseases enter the clinical arena through different leading domains before progressively involving the others. As visually synthesized in Figure 1, Alzheimer’s disease is predominantly cognition-led; frontotemporal dementia is behaviour-led; Parkinsonian syndromes are motor-led; dementia with Lewy bodies is multidomain from early stages. This trajectory-based framework has direct implications not only for diagnosis, but for therapeutic reasoning, iatrogenic harm prevention, deprescribing, and palliative planning [5,6,7,8].

## 2. Why Clinical Trajectories Matter in Older Adults

The concept of clinical trajectory is especially relevant in geriatrics because ageing modifies both the expression and the meaning of disease. Biological ageing, reduced physiological reserve, cumulative brain pathology, systemic inflammation, vascular burden, and chronic medication exposure all contribute to a phenotype in which neurological symptoms are distributed across interconnected functional domains rather than neatly confined to one system. In older adults, symptom boundaries are porous: a single clinical presentation may reflect multiple simultaneous pathological processes, none of which is fully captured by any single diagnostic label [1,3,7].

A trajectory-based perspective offers four main clinical advantages. First, it captures heterogeneity within diagnostic categories. Alzheimer’s disease may begin with language, visuospatial, dysexecutive, or behavioural features; Parkinson’s disease may remain tremor-dominant for years or evolve early into a more axial and cognitively vulnerable phenotype; frontotemporal dementia may present as behavioural variant disease, primary progressive aphasia, or an overlap syndrome; and dementia with Lewy bodies may declare itself through cognitive fluctuation, hallucinations, REM sleep behaviour disorder, or autonomic dysfunction. Static labels cannot fully represent this diversity; trajectories can [8,9,10,11,12]. The dynamic and reversible nature of frailty further strengthens the case for a trajectory-based perspective. In a nationally representative longitudinal cohort, sustained frailty remission over three consecutive assessment waves was associated with a 34% reduction in incident dementia risk compared with persistent frailty or pre-frailty, with the protective effect most pronounced in younger-old adults below 80 years and in male participants [13]. This finding positions frailty not merely as a static comorbidity but as a modifiable trajectory state—one whose improvement or deterioration has direct implications for cognitive outcomes. Beyond physical frailty, social frailty—defined by vulnerability in social resources and connections—has also emerged as an independent predictor of incident dementia. A 12-year longitudinal study of community-dwelling adults found that socially frail individuals carried approximately 47% higher dementia risk compared with non-frail peers, independent of physical and psychological frailty, with low financial satisfaction, infrequent social contact, and reduced participation in social activities as the key predictive components [14]. Together, these data argue that the clinical trajectory of neurodegenerative disease is shaped not only by its dominant pathology but by the modifiable frailty state of the patient in whom it unfolds. A critical practical clarification applies to the oldest-old and to patients with significant sensory or musculoskeletal comorbidity: sphere-entry identification is not always unambiguous, and the framework does not require it to be. In patients over 85 with multimorbidity, symptoms across the three spheres may emerge simultaneously or be partially masked by arthritis, hearing loss, visual impairment, or deconditioning. In such cases, the appropriate clinical response is not to force a single dominant sphere label but to recognize multidomain convergence as a legitimate trajectory pattern—one exemplified by dementia with Lewy bodies and by advanced mixed dementia—and to apply the framework iteratively, reassessing sphere dominance across serial encounters rather than at a single assessment point. The goal of the framework is to inform clinical reasoning and anticipate therapeutic trade-offs, not to generate a rigid classification. Second, trajectories help explain diagnostic ambiguity across diseases. What often distinguishes one disease from another is not merely the presence of a symptom, but the way that symptom emerges, combines with others, and evolves through time. Third, trajectories have direct therapeutic relevance because drugs rarely act on one domain alone in older adults. Dopaminergic therapies may improve movement while worsening hallucinations or impulse control; antipsychotics may reduce distress while worsening gait, swallowing, or mortality risk. Fourth, trajectories allow earlier recognition of preclinical and transitional syndromes, consistent with recent work on motoric-cognitive risk, sleep disorders across the parkinsonism continuum, and neuromuscular ageing [5,6,7,9,10,11,12].

## 3. The Three-Sphere Framework and Its Biological Underpinnings

The three-sphere framework proposed in this review provides a clinically intuitive and biologically plausible way of reading neurodegenerative disease in later life. Each sphere corresponds to a dominant domain of dysfunction, but the spheres are not independent compartments. They are interconnected dimensions of the same evolving disease process, and their interaction is often the key determinant of clinical burden and therapeutic risk [4,5,7].

### 3.1. Cognitive Sphere

The cognitive sphere includes episodic memory, executive function, language, visuospatial processing, attention, processing speed, and social cognition. Different disorders privilege different networks: Alzheimer’s disease typically begins with episodic memory impairment, with low free recall not normalized by cueing, reflecting hippocampal dysfunction, and later extends to language, visuospatial, and executive domains; dementia with Lewy bodies more often shows early attentional fluctuation, visuospatial dysfunction, and executive inefficiency; Parkinson’s disease dementia usually shows a frontostriatal-posterior cortical pattern; and frontotemporal dementia may spare traditional memory measures early while profoundly disturbing executive and social cognition [4,8,9,10,11,12].

### 3.2. Behavioural-Neuropsychiatric Sphere

The behavioural-neuropsychiatric sphere includes depression, anxiety, apathy, psychosis, agitation, aggression, disinhibition, sleep disorders, and other behavioural and psychological symptoms of dementia (BPSD), which occur in over 90% of individuals with dementia and frequently cause more distress than cognitive deficits alone. This sphere has strong biological roots in neurotransmitter dysregulation, serotonergic, dopaminergic, cholinergic, and noradrenergic, and in frontolimbic network disconnection, and is deeply shaped by acute stressors such as infection, pain, hospitalization, or sleep deprivation. In frontotemporal dementia, it is often the dominant expression of disease; in dementia with Lewy bodies, it is inseparable from cognitive and autonomic dysfunction; in Alzheimer’s disease, it becomes progressively more relevant as the disease broadens beyond memory; and in Parkinsonian syndromes, it is entangled with dopaminergic treatment and cortical spread [8,11,12].

### 3.3. Extrapyramidal-Motor Sphere

The extrapyramidal-motor sphere includes bradykinesia, rigidity, tremor, gait impairment, postural instability, axial dysfunction, hypomimia, hypophonia, freezing, and falls. The neuropathological substrate virtually always involves degeneration of the nigrostriatal dopaminergic system, but the underlying molecular pathology varies: alpha-synuclein accumulation in Parkinson’s disease and dementia with Lewy bodies; tau pathology in progressive supranuclear palsy and corticobasal degeneration; or mixed pathologies. This sphere is the dominant point of entry in Parkinson’s disease and related synucleinopathies, but extrapyramidal signs also emerge in dementia with Lewy bodies, frontotemporal lobar degeneration syndromes, vascular cognitive impairment, and even advanced Alzheimer’s disease. In older adults, motor decline rapidly translates into falls, immobility, hospitalization, and mortality [7,9,10,11,12,15].

### 3.4. Dynamic Interactions Between Spheres and the Delirium Axis

The central strength of the three-sphere model is that it emphasizes interaction rather than separation. A patient with worsening cognition may become behaviourally dysregulated because of disorientation or impaired impulse control. Behavioural symptoms may worsen motor performance through poor sleep, inattention, apathy, or excessive psychotropic burden. Motor dysfunction may accelerate cognitive and affective decline by reducing activity, social exposure, autonomy, and confidence. Dopaminergic medications improve motor function through nigrostriatal pathway stimulation but simultaneously affect mesolimbic and mesocortical dopamine pathways, potentially precipitating hallucinations, psychosis, and impulse control disorders, with dopamine agonists carrying two to four times higher risk than levodopa. Antipsychotics, conversely, block D2 receptors to reduce psychosis but cause or exacerbate extrapyramidal symptoms, increasing fall risk, aspiration pneumonia risk, and mortality [4,9,10,11,12]. Superimposed on this three-sphere model, delirium must be recognized as a fourth disruptive axis. Delirium is an acute, fluctuating disturbance in attention and awareness that affects 15–50% of hospitalized older adults and is particularly common in those with pre-existing neurodegeneration. It should not be interpreted as simply another behavioural symptom or as a generic marker of worsening dementia; it is better understood as an acute disruptive process that can transiently deform all three spheres at once, mimicking disease progression while in fact representing a potentially reversible destabilization. Medication-induced delirium is especially relevant given the polypharmacy burden of these patients: implicated agents include anticholinergics, benzodiazepines, opioids, H2-receptor antagonists, diphenhydramine, and sedative-hypnotics [16,17].

## 4. Disease-Specific Clinical Trajectories

### 4.1. Alzheimer’s Disease: A Cognition-Led Trajectory That Progressively Broadens

**Figure 2:**
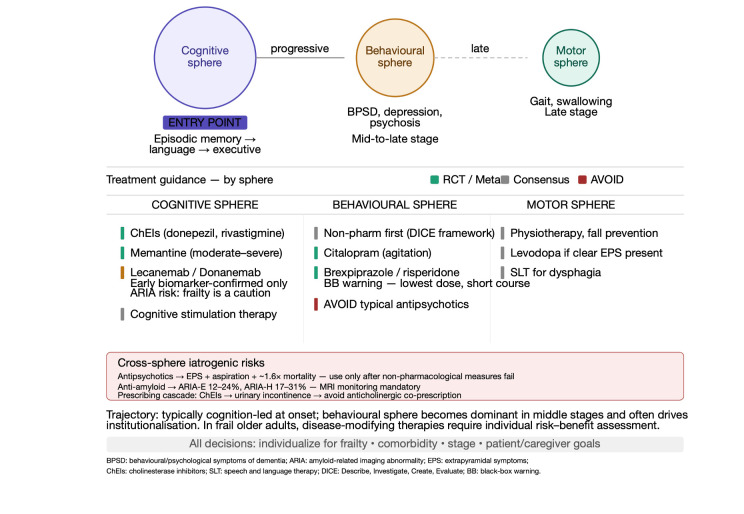
Therapeutic scheme for Alzheimer’s disease organized by symptom sphere. Cognitive problems are addressed with donepezil, rivastigmine, or memantine; extrapyramidal symptoms, when present, with levodopa/benserazide or selegiline; BPSD with typical and atypical antipsychotics and antidepressants; anxiety and psychosis require avoidance of dopaminergic agents and preferential use of quetiapine or clozapine.

Each of the following disease-specific sections is organized according to a consistent trajectory logic: the dominant entry sphere through which the disease typically presents, the characteristic pattern of sphere transitions as the disease progresses, the main iatrogenic risks arising from cross-sphere drug interactions, and the practical management implications of that trajectory in older adults with multimorbidity. Alzheimer’s disease remains the most recognizable cognition-led neurodegenerative disorder in later life, accounting for 60–70% of dementia cases and typically progressing over 8–12 years from initial symptom onset to death. In older adults, it unfolds as a longitudinal process in which the cognitive sphere predominates early, while behavioural-neuropsychiatric features, sleep disruption, frailty, and motor signs progressively gain relevance and often determine the real burden of disease (Figure 2) [4,18]. The biomarker era has transformed the conceptualization of Alzheimer’s disease. The 2024 revised Alzheimer’s Association Workgroup criteria define the disease as a biological process beginning with the appearance of amyloid plaques and neurofibrillary tangles while individuals are still asymptomatic. Core 1 biomarkers, amyloid PET, CSF amyloid-β42/40 ratio, and plasma phosphorylated tau-217 (p-tau217) become abnormal early and are sufficient to establish a biological diagnosis. Core 2 biomarkers (tau PET, CSF p-tau181) become abnormal later and provide prognostic information. Importantly, only approximately one-third of amyloid-positive individuals follow the predicted amyloid cascade trajectory, with substantial proportions showing resilience or copathologic patterns, a reality particularly relevant in older adults in whom mixed pathologies are common [4,18,19,20]. Therapeutically, Alzheimer’s disease now spans two broad phases. The long-standing phase of symptomatic treatment relies on cholinesterase inhibitors, donepezil, rivastigmine, and galantamine, which partially compensate for cholinergic neuronal loss. The Alzheimer’s Disease Assessment Scale–Cognitive subscale (ADAS-Cog) is a validated 70-point instrument measuring multiple cognitive domains including memory, language, praxis, and orientation, where higher scores reflect greater impairment; the Alzheimer’s Disease Cooperative Study–Activities of Daily Living scale (ADCS-ADL) quantifies functional independence across basic and instrumental activities, with higher scores indicating better function. Meta-analyses demonstrate approximately 2.4-point improvement on the 70-point ADAS-Cog scale over 6–12 months, with benefits extending to activities of daily living, global function, and behavioural symptoms, and long-term observational data suggesting 27–42% reduction in mortality risk over 2–8 years. The second phase is the era of selective disease-modifying intervention in early, biomarker-confirmed disease: in CLARITY-AD, lecanemab slowed cognitive and functional decline by 27% over 18 months; in TRAILBLAZER-ALZ 2, donanemab slowed decline by 35–36% under a treat-to-clear model. Both agents carry significant amyloid-related imaging abnormality (ARIA) risk (ARIA-E in 12.6% and 24.0%, respectively; ARIA-H in 17.3% and 31.4%), and their modest clinical benefit, substantial cost, and safety profile necessitate careful shared decision-making, particularly in frail older adults with multimorbidity. [21,22] Beyond safety, the current cost of anti-amyloid therapies—exceeding USD 26,000 per year for lecanemab alone, excluding MRI monitoring and infusion costs—represents a structural barrier to equitable access even within high-income healthcare systems, and raises serious questions of cost-effectiveness in patients where frailty, multimorbidity, and limited life expectancy may substantially alter the benefit-to-burden ratio. The three-sphere framework explicitly guides the identification of patients in whom disease-modifying therapy is appropriate (early biomarker-confirmed AD, MMSE ≥ 22, absence of anticoagulation, no significant cerebrovascular disease, frailty-stratified risk assessment) versus those in whom trade-offs are unfavourable. As Alzheimer’s disease advances, the behavioural-neuropsychiatric sphere becomes increasingly relevant. Depression, irritability, apathy, anxiety, psychosis, circadian disruption, and agitation often become the real drivers of caregiver exhaustion and institutionalization. Non-pharmacological interventions represent first-line treatment for BPSD. When pharmacological management is necessary, the strongest evidence for agitation supports citalopram; brexpiprazole and risperidone are the only atypical antipsychotics with regulatory approval for this indication. Atypical antipsychotics carry black-box warnings for increased mortality (1.6–1.7-fold increase) and should be used at the lowest doses for the shortest durations. In practical terms, Alzheimer’s disease usually begins as a cognition-led trajectory but eventually becomes a multidomain syndrome, and management must evolve accordingly [4,16,17,23].

### 4.2. Frontotemporal Dementia: A Behaviour-Led Trajectory with Network and Phenotypic Diversity

Frontotemporal dementia provides perhaps the clearest example of why symptom trajectories matter more than diagnostic shorthand. In its behavioural variant, representing 50–70% of cases and typically presenting in the sixth to seventh decade, the disease often enters the clinical field not through forgetfulness but through altered personality, social misconduct, emotional blunting, impulsivity, ritualization, dietary change, loss of empathy, or apparently inexplicable relational deterioration. This behaviour-led trajectory is frequently misread as psychiatric, moral, or interpersonal rather than neurological, leading to initial psychiatric referral in up to 44% of cases [8].

**Figure 3:**
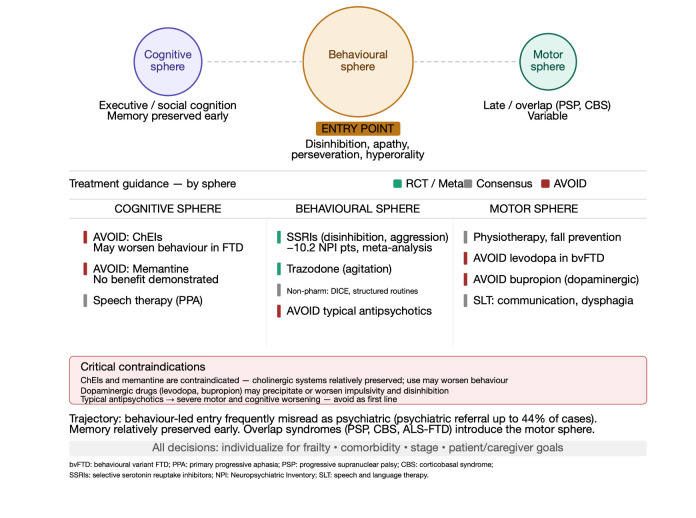
Therapeutic scheme for frontotemporal dementia organized by symptom sphere. Behavioural-psychological symptoms represent the dominant sphere and are treated with typical and atypical antipsychotics and antidepressants; cognitive problems with memantine; extrapyramidal symptoms require avoidance of levodopa/benserazide, selegiline, and dopaminergic antidepressants such as bupropion; anxiety and psychosis with quetiapine, clozapine, or olanzapine.

This predominance of the behavioural-neuropsychiatric sphere reflects the preferential vulnerability of frontal and anterior temporal networks involved in motivation, inhibition, social cognition, affective resonance, and self-regulation. Executive dysfunction and social-cognitive failure typically predominate over episodic memory impairment, especially early in the course. The neuropathological substrate is heterogeneous: approximately 40–50% show tau pathology (Pick’s disease, corticobasal degeneration, progressive supranuclear palsy), and 40–50% show TDP-43 proteinopathy. Approximately 20–30% of cases are familial, with mutations in C9orf72, GRN, and MAPT genes accounting for most genetic cases. Primary progressive aphasias and overlap syndromes such as progressive supranuclear palsy, corticobasal syndrome, and FTD-ALS further illustrate that the boundaries between behavioural, cognitive, and motor trajectories are porous [8]. These distinctions are therapeutically decisive (Figure 3). Selective serotonin reuptake inhibitors and trazodone remain the most rational symptomatic options for behavioural symptoms, with meta-analysis demonstrating a mean reduction of 10.17 points on the Neuropsychiatric Inventory with SSRI treatment. SSRIs show particular benefit for disinhibition, irritability, aggression, perseverative behaviours, and hyperorality. Crucially, cholinesterase inhibitors are not appropriate in frontotemporal dementia and may worsen behavioural abnormalities, reflecting the relative preservation of cholinergic systems in FTD compared to Alzheimer’s disease. Memantine similarly does not improve or delay progression [8].

### 4.3. Parkinson’s Disease and Parkinson’s Disease Dementia: A Motor-Led Trajectory with Progressive Multidomain Involvement

**Figure 4:**
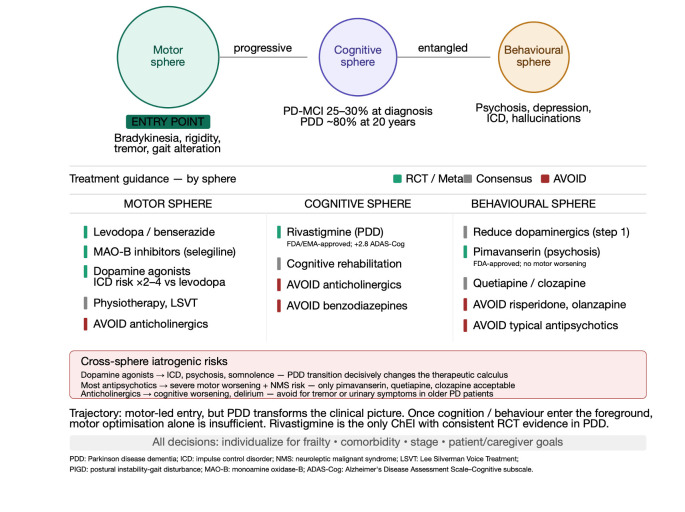
Therapeutic scheme for Parkinson’s disease and Parkinson’s disease dementia organized by symptom sphere. Extrapyramidal symptoms are treated with levodopa/benserazide, dopamine agonists, or selegiline; cognitive problems with rivastigmine; BPSD with atypical antipsychotics and antidepressants; anxiety and psychosis with quetiapine or clozapine, with careful stepwise reduction of dopaminergic agents.

Parkinson’s disease remains the prototype of a motor-led trajectory, yet it is not adequately defined by its motor syndrome alone. Bradykinesia, rigidity, tremor, gait alteration, and postural instability remain the cardinal entry points, but the disease progressively extends into cognition, behaviour, sleep, autonomic regulation, and mood (Figure 4). The clinical heterogeneity of motor presentation is substantial: the postural instability-gait disturbance (PIGD) phenotype is associated with faster motor progression, greater severity of non-motor symptoms, and worse prognosis, while motor subtypes are not static and may evolve with disease progression [9,10]. Mild cognitive impairment (PD-MCI) affects 25–30% of patients at diagnosis, and dementia (PDD) develops in approximately 80% of patients who survive 20 years, with a cumulative incidence of approximately 10% per year. The cognitive profile in PDD emphasizes executive dysfunction, attention deficits, and visuospatial impairment more than memory deficits, reflecting frontostriatal circuit dysfunction and posterior cortical involvement, consistent with recent work on motoric-cognitive vulnerability, sleep disorders across the parkinsonism continuum, and neuromuscular ageing [5,6,7,9,10]. The transition from Parkinson’s disease to Parkinson’s disease dementia is a qualitative turning point in trajectory. Once cognition and behaviour enter the foreground, motor optimization alone is no longer sufficient and may become actively harmful. Dopaminergic treatment remains essential for mobility, yet dopamine agonists carry two to four times higher risk of impulse control disorders compared to levodopa. For management of psychosis in Parkinson’s disease, dopaminergic medications should be reduced stepwise before adding pimavanserin—the only FDA-approved agent for Parkinson’s disease psychosis, as it does not worsen motor function—or quetiapine or clozapine. Typical antipsychotics and most atypical antipsychotics (risperidone, olanzapine, aripiprazole) are contraindicated due to severe motor worsening and increased risk of neuroleptic malignant syndrome. Rivastigmine is the only cholinesterase inhibitor with consistent positive outcomes in randomized controlled trials for PDD (FDA/EMA-approved), demonstrating 2.80-point improvement on ADAS-Cog and 2.50-point improvement on ADCS-ADL compared to placebo [6,9,10].

### 4.4. Dementia with Lewy Bodies: Early Multidomain Convergence as a Distinctive Trajectory

**Figure 5:**
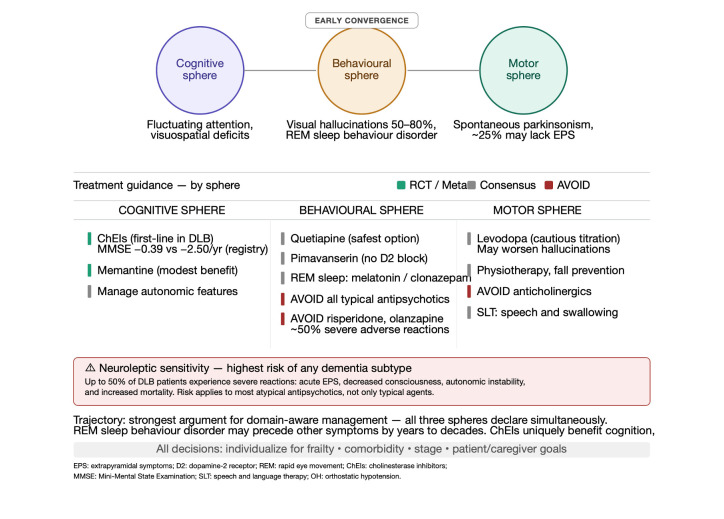
Therapeutic scheme for dementia with Lewy bodies organized by symptom sphere. Cognitive problems are treated with donepezil, rivastigmine, or memantine; extrapyramidal symptoms with levodopa/benserazide or selegiline; BPSD with atypical antipsychotics and antidepressants; anxiety and psychosis with quetiapine or clozapine, with strict avoidance of typical antipsychotics and careful management of neuroleptic sensitivity.

If Alzheimer’s disease is typically cognition-led and Parkinson’s disease motor-led, dementia with Lewy bodies is best understood as a disorder of early multidomain convergence (Figure 5). In DLB, cognition, behaviour, sleep, motor features, and autonomic dysfunction often become clinically visible in close temporal proximity rather than in an orderly sequence. The cognitive profile features fluctuating attention, executive inefficiency, and visuospatial impairment rather than predominantly amnestic syndrome. Recurrent visual hallucinations (50–80% of patients) and REM sleep behaviour disorder (50–75%, frequently preceding other symptoms by years to decades) are core diagnostic features. Spontaneous parkinsonism develops in most patients, though up to 25% never develop extrapyramidal signs, and tremor is less prominent than in Parkinson’s disease [11,12]. This pattern makes DLB one of the strongest arguments for domain-aware and trajectory-sensitive management. These patients are particularly vulnerable to neuroleptic sensitivity: up to 50% experience severe adverse reactions, marked worsening of extrapyramidal symptoms, decreased consciousness, autonomic instability, and increased mortality following antipsychotic exposure. When antipsychotic treatment is absolutely necessary, quetiapine represents the safest option; pimavanserin is a promising alternative given its lack of dopamine receptor antagonism. Conversely, cholinesterase inhibitors represent first-line pharmacological treatment and may simultaneously support cognition, reduce cognitive fluctuations, and benefit hallucinations and apathy. The Mini-Mental State Examination (MMSE) is a widely used 30-point cognitive screening instrument assessing orientation, registration, attention, recall, language, and visuoconstructive ability; scores of 24–30 indicate no or minimal impairment, 18–23 mild impairment, 10–17 moderate impairment, and below 10 severe impairment. Long-term data from Swedish registries demonstrate treated patients show lower MMSE decline (−0.39 vs. −2.50 points/year in non-users) and reduced first-year mortality (HR 0.66). Autonomic features, including orthostatic hypotension, constipation, and urinary dysfunction, and REM sleep behaviour disorder further reinforce the multidomain nature of the disorder and the need for integrated care [11,12].

### 4.5. Vascular Cognitive Impairment and Mixed Dementia: Heterogeneous Trajectories Modified by Burden, Reserve, and Copathology

Vascular cognitive impairment and dementia represent a heterogeneous group of disorders rather than one canonical course. Mixed dementia, vascular pathology coexisting with Alzheimer’s disease, Lewy body, or TDP-43 pathology, is actually more common than pure vascular dementia, occurring in 30–40% of dementia cases. The prevalence of mixed pathology in aged brains is substantially higher than clinical recognition would suggest. Autopsy data from 13 community-based cohorts comprising over 6000 individuals revealed LATE neuropathologic change—limbic-predominant age-related TDP-43 encephalopathy—in approximately 40% of participants, with the condition present in roughly 55% of brains with severe Alzheimer’s pathology and in 27% of brains with no detectable amyloid plaques [24]. These findings have a direct clinical implication for the trajectory-based framework: in the oldest-old, cognitive phenotype frequently reflects the combined expression of two or more partially overlapping pathological processes, none of which is fully captured by any single biomarker or diagnostic label. The biological reductionism of current biomarker-based staging models—however useful in research settings—systematically underestimates the degree of pathological heterogeneity encountered in geriatric clinical practice, reinforcing the argument that domain-sensitive clinical frameworks are not merely complementary to biomarker diagnosis but operationally necessary in the most complex patients. The three-sphere framework is deliberately pathology-agnostic in this context: it operates on clinically observable domain dominance rather than neuropathological substrate, which is rarely accessible in vivo. Even when two or more pathological processes coexist and interact, the clinician can still identify which sphere is currently generating the most therapeutic complexity and apply the framework’s cross-sphere risk logic accordingly. Trajectory heterogeneity in mixed dementia argues for iterative, domain-sensitive clinical reassessment—precisely what the three-sphere approach is designed to support—rather than for abandoning structured reasoning in favour of pure empiricism. The pathophysiological mechanisms include direct tissue loss from infarcts and haemorrhages, white matter injury from chronic hypoperfusion and blood–brain barrier disruption, neurovascular unit dysfunction, neuroinflammation, oxidative stress, and interaction with neurodegenerative pathologies, with evidence that vascular pathology promotes amyloid deposition and tau pathology [15,25]. Some vascular trajectories are stepwise, particularly after clinically evident strokes or recurrent infarcts; others are more insidious, with progressive executive dysfunction, slowing, attentional impairment, gait disturbance, and apathy gradually emerging in parallel. In small-vessel disease, the cognitive sphere is often more dysexecutive-subcortical than amnestic, while the motor sphere may become prominent early through gait impairment and instability. The SPRINT MIND trial demonstrated that intensive systolic blood pressure lowering to <120 mmHg reduced the combined incidence of mild cognitive impairment and dementia by 19%: the strongest clinical evidence for dementia prevention through any single intervention to date. No FDA-approved medications exist specifically for pure vascular dementia; the therapeutic emphasis remains on prevention and vascular burden reduction, with biomarker assessment for Alzheimer’s copathology guiding use of cholinesterase inhibitors in mixed dementia cases [15,25].

## 5. Therapeutic Implications of Cross-Sphere Interactions

**Figure 6:**
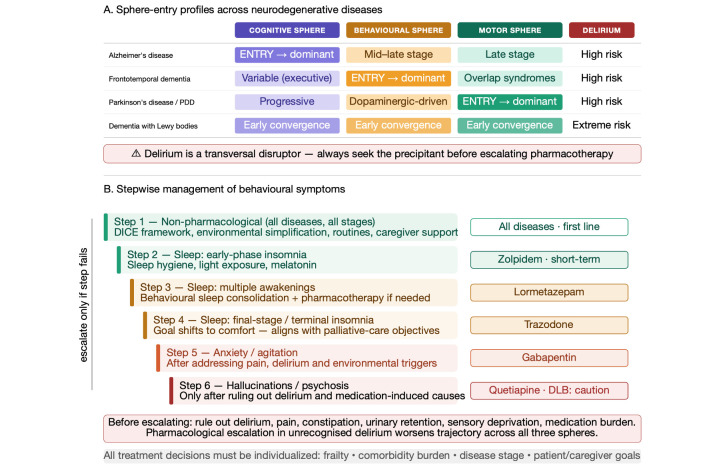
Comprehensive three-sphere trajectory map for neurodegenerative diseases in older adults. The figure integrates five disease-specific trajectories (Alzheimer’s disease, frontotemporal dementia, Parkinson’s disease/PDD, dementia with Lewy bodies, and vascular-mixed dementia) across the cognitive, behavioural-neuropsychiatric, and motor spheres. Entry points, progression patterns, and critical cross-sphere therapeutic trade-offs are displayed alongside frailty and iatrogenic risk overlays. The delirium axis is represented as a transversal vulnerability layer that intersects all three spheres and all disease trajectories. This integrated view is intended to support domain-aware clinical reasoning and multidisciplinary team communication in real-world geriatric practice

The three-sphere framework becomes most clinically useful when translated into treatment logic. In older adults, pharmacological choices rarely act on one domain alone. Treatment should begin by identifying the currently dominant sphere, then anticipating cross-sphere trade-offs, then matching goals to stage. A patient with Alzheimer’s disease may be primarily destabilized by nocturnal behavioural disorder rather than memory decline; a patient with Parkinson’s disease may be more threatened by hallucinations, orthostatic hypotension, and falls than by bradykinesia; a patient with frontotemporal dementia may present clinically through caregiver collapse rather than through measurable cognitive deterioration [4,9,10,11,12,23]. Cross-sphere trade-offs are one of the strongest clinical messages of the model. Dopaminergic drugs may restore mobility but worsen hallucinations, impulsivity, and sleep fragmentation. Antipsychotics may suppress psychosis or agitation but impair swallowing, mobility, orthostatic resilience, and arousal, with number needed to harm analyses estimating approximately nine excess deaths per 100 patients treated with haloperidol for 180 days, four with risperidone, three with olanzapine, and two with quetiapine. Cholinesterase inhibitors may improve attention and hallucinations in one disorder while contributing to gastrointestinal distress, bradycardia, sleep disruption, or urinary urgency in another. Sedative strategies may transiently reduce distress but at the cost of falls, aspiration, immobility, and delirium. To prescribe without anticipating these interactions is to risk accelerating the trajectory one is trying to control [9,10,11,12,16,17,21,22,23]. Treatment goals must be stage-sensitive. In early disease, priorities include biological clarification, disease-modifying opportunities where appropriate, preservation of instrumental autonomy, and support for future planning. In middle stages, management shifts toward behavioural containment, fall prevention, sleep stabilization, caregiver support, and rational simplification of therapy. In advanced stages, the dominant goal becomes reduction of suffering, avoidance of burdensome interventions, and preservation of dignity. A trajectory-based therapeutic strategy must also remain multidisciplinary by design, because no pharmacological plan is stable if it ignores sleep, pain, constipation, dysphagia, sensory loss, social isolation, caregiver depletion, or rehabilitation needs [9,10,11,12,15,23,26,27]. The cross-sphere therapeutic logic described in this section, including sphere-entry profiles across major disease trajectories, critical iatrogenic trade-offs, and the stepwise approach to behavioural management, is comprehensively illustrated in Figure 6.

## 6. Delirium, Polypharmacy, and Deprescribing

Among all the processes that can abruptly alter the course of neurodegenerative disease in older adults, delirium is one of the most consequential and most frequently misread. When delirium is mistaken for advancing dementia, clinicians may escalate antipsychotics, benzodiazepines, sedatives, or other centrally acting drugs without identifying the true precipitant. Common triggers include infection, dehydration, pain, constipation, urinary retention, metabolic disturbance, sleep disruption, sensory deprivation, immobilization, hospitalization, surgery, and medication exposure; agents particularly associated with delirium in this setting include anticholinergics, benzodiazepines, opioids, H2-receptor antagonists, sedative-hypnotics, and antipsychotics [16,17]. Polypharmacy is structurally embedded in the way these patients are managed. Patients with Alzheimer’s disease carry an average of 4–6 chronic conditions and 5–8 medications. Despite guidelines recommending deprescribing, patients with incident Alzheimer’s disease demonstrate increased overall medication use in the year following diagnosis, driven primarily by initiation of cholinesterase inhibitors and CNS-active medications; CNS-active medication use increases by 15–20% in the year surrounding diagnosis. Among Medicare beneficiaries with Alzheimer’s disease, 76–78% continue receiving high-risk medications like antipsychotics, sedative-hypnotics, and strong anticholinergics despite their established risks [3,16,17,23]. The prescribing cascade—in which an adverse drug reaction is misidentified as a new medical condition and treated with a second medication—represents one of the most concrete and quantifiable manifestations of polypharmacy-driven iatrogenic harm in this population. A well-documented cascade involves the co-prescription of anticholinergic agents to manage cholinesterase inhibitor-induced urinary incontinence; in a large Medicare cohort of nursing home residents with severe dementia, approximately one in seven patients receiving cholinesterase inhibitors was also prescribed a strong anticholinergic agent, with non-geriatrician prescribers and polypharmacy independently associated with higher co-prescribing rates [28]. A second high-priority cascade involves the initiation of antiparkinsonian agents following antidopaminergic drug exposure—including antipsychotics and metoclopramide—in patients with Alzheimer’s disease, illustrating how drugs introduced to manage behavioural sphere symptoms may generate iatrogenic motor sphere involvement [29]. Population-based data across multiple cascade types confirm that diuretics following calcium channel blockers, anticholinergics following cholinesterase inhibitors, and dopaminergic agents following antipsychotics each occur with clinically meaningful frequency in older adults with dementia, with female sex and advancing comorbidity burden as consistent risk factors [30]. These cascades are precisely what the three-sphere framework is designed to expose: when a clinician treats agitation without anticipating motor consequences, or manages motor symptoms without tracking cognitive and behavioural downstream effects, the conditions for cascade generation are systematically created. Deprescribing is therefore a core therapeutic act in precision geriatric neurology. A practical framework involves four steps: identifying targets by eliciting medication-related goals from patients and caregivers; developing a tapering plan with specific schedules and monitoring parameters; educating patients and caregivers about the rationale; and providing close follow-up. Priority targets include medications with strong evidence of harm (anticholinergics, benzodiazepines, antipsychotics), medications for primary prevention with limited benefit given life expectancy (statins, bisphosphonates), and medications that may be causing symptoms, like akathisia from SSRIs or urinary urgency from cholinesterase inhibitors. The D-PRESCRIBE-AD trial underscores how difficult this work is in practice, but also why it remains clinically essential [23].

## 7. Non-Pharmacological and Multidisciplinary Care

One of the most important practical consequences of the three-sphere model is that it repositions non-pharmacological care from supportive background to therapeutic foreground. Behavioural escalation may arise from pain, boredom, sensory overload, loneliness, disrupted routine, constipation, fear, poor communication, or sleep fragmentation. Gait deterioration may be amplified by deconditioning, inappropriate footwear, poor lighting, sedative burden, or environmental clutter. If these contributors are not addressed, pharmacological treatment is asked to solve problems it was never designed to solve [11,26,31]. In disorders where the behavioural sphere is prominent, structured behavioural assessment using the DICE framework (Describe, Investigate, Create, Evaluate) is essential. Environmental simplification, reduction of noise, improved lighting, preservation of routines, orientation support, and meaningful activities tailored to previous identity and preserved abilities can all reduce behavioural distress. Sensory interventions with consistent evidence include music therapy (effect size 0.2–0.5 for depression and apathy), massage and touch therapy (SMD −0.75 for agitation, rising to −0.91 when combined with music), and pet therapy (SMD −0.47). Recent umbrella-review evidence continues to support non-pharmacological interventions as first-line strategies for BPSD. A critical caveat applies to all of these recommendations: their feasibility is inseparable from the availability of caregiver support, professional training, and the material resources of the caregiving context. For a sole family caregiver managing a patient with advanced dementia at home without professional assistance, structured activity programmes, music therapy sessions, or pet-assisted interventions may be operationally inaccessible. In such circumstances, the clinical priority shifts toward caregiver burden assessment and respite care provision—evidence-based interventions that must precede or accompany any non-pharmacological recommendation. The DICE framework explicitly includes a caregiver resource assessment as its first step, and this should be understood as a non-negotiable prerequisite, not an afterthought [11,26,31]. Rehabilitation acquires particular relevance because the motor sphere is both a source of disability and a point of intersection between domains. Meta-analytic evidence demonstrates that exercise significantly reduces neuropsychiatric symptoms (mean difference −5.28, 95% CI −9.46 to −1.11) and depression in dementia. Aerobic exercise performed three to five times weekly shows the most consistent benefits; strength training significantly reduces depression and behavioural problems in patients with mobility limitations. In Parkinson’s disease, physical therapy, Lee Silverman Voice Treatment (LSVT LOUD) for speech, and cueing-based gait training improve multiple domains simultaneously [6,7,9,15,32]. Caregivers often detect changes across spheres long before those changes are visible in brief clinical encounters, and caregiver burden itself becomes a clinical variable that can amplify crisis-driven prescribing or institutional transition. Caregiver-centred interventions, including psychoeducation, skills training, and activity planning, reduce both BPSD frequency and severity (effect size 0.34) and caregiver burden (effect size 0.15). Comprehensive dementia care management models incorporating care coordination, medication optimization, and integrated pharmacological and non-pharmacological interventions have demonstrated significant reductions in behavioural symptoms in large-scale randomized trials [26,31].

## 8. Prognostic and Palliative Trajectories

Prognosis in neurodegenerative disease is better interpreted as a longitudinal clinical process than as a single prediction delivered at diagnosis. Median survival from dementia diagnosis varies by dementia type: Alzheimer’s disease 5–6 years, frontotemporal dementia 5 years, vascular dementia and dementia with Lewy bodies approximately 4 years, with individual variability from less than 1 year to more than 10 years. The strongest predictors of mortality are cognitive severity (MMSE), functional status (ADL dependence), and dementia type; male sex, older age at diagnosis, higher comorbidity burden, and neuropsychiatric symptom severity are further independent predictors. Several validated prognostic models can support, but not replace, individualized clinical judgment [25,33,34]. The shift from disease-oriented to comfort-oriented treatment is rarely a single event. Goals of care usually evolve gradually and must be revisited repeatedly. Advanced dementia-specific indicators warranting consideration of goals-of-care discussions include recurrent hospitalizations; progressive dependence; severe dysphagia; nutritional decline with albumin <3.5 g/dL or unintentional weight loss; profound behavioural distress; pressure ulcers; and persistent low-arousal states. When these emerge, the disease may have reached a stage where further diagnostic escalation or preventive pharmacology offers little meaningful gain. Specialist palliative care referral criteria are now being defined more explicitly, and major palliative care guidance in neurology supports earlier and iterative care planning rather than waiting for a terminal threshold [26,27,33,34]. Advance care planning is a core longitudinal intervention that preserves personhood across the trajectory of progressive loss. Key content includes eliciting values and preferences, goals of care, specific intervention preferences (hospitalization, tube feeding, antibiotics, CPR, mechanical ventilation), and surrogate decision-maker designation. Observational studies consistently show no benefits of tube feeding for survival, aspiration pneumonia prevention, nutritional status, or comfort in advanced dementia; careful hand-feeding for comfort is preferred. Patients with advance directives have better palliative care outcomes: less tube feeding, fewer hospitalizations near end of life, and greater hospice enrolment. ACP interventions increase documentation of advance directives (RR 1.23) and discussions about goals of care (RR 1.33), and may increase concordance with goals of care (RR 1.39) [26,27,34,35].

## 9. Discussion

The main argument of this review is that neurodegenerative diseases in older adults are better understood as dynamic multidomain trajectories than as static diagnostic labels. This does not weaken diagnosis; it contextualizes it. Alzheimer’s disease, frontotemporal dementia, Parkinsonian syndromes, dementia with Lewy bodies, and vascular-mixed dementias remain biologically and clinically distinct, but their relevance in real-world care depends on how they move through cognition, behaviour, and mobility across time [4,5,6,7,8,9,10,11,12,15]. This approach is particularly valuable in geriatrics because it aligns clinical reasoning with the reality of older patients: interacting proteinopathies, vascular burden, inflammation, frailty, sensory loss, sleep disruption, social vulnerability, and high medication load. For this reason, the clinical phenotype is often more complex than the diagnostic label suggests, and a trajectory-based framework preserves disease specificity while allowing clinicians to reason in a more realistic, domain-sensitive way [1,2,3,4,12,15]. The model also has a strong anti-iatrogenic function. If a clinician treats agitation without considering gait and swallowing, or treats gait without considering hallucinations and sleep, the result may be apparent symptom control but true trajectory worsening. In older adults, the most meaningful therapeutic harm often arises not from dramatic mistakes but from repeated small decisions made without a multidomain perspective. The three-sphere framework helps expose those decision points, links biomarker-driven advances back to bedside judgment, and supports earlier recognition of the critically important transition from disease-modifying to comfort-oriented goals of care [9,10,11,12,15,16,17,18,21,22,23].

### Strengths and Limitations

Strengths of this review include its comprehensive scope, the integration of epidemiology, molecular pathophysiology, biomarker-based diagnosis, pharmacotherapy, non-pharmacological interventions, and palliative care across the full spectrum of neurodegenerative diseases, and the clinical applicability of the three-sphere framework as a practical organizing structure for real-world decision-making in complex older adults. The review incorporates recent high-quality evidence including anti-amyloid therapy trials, revised biomarker-based diagnostic criteria, and systematic reviews and meta-analyses of interventions. Limitations include the narrative format with potential for selection bias in evidence presentation; the rapidly evolving nature of dementia therapeutics and diagnostics; limited generalizability from evidence predominantly derived from high-income countries; and the unavoidable gap between a general framework and the full complexity of individual patients. Many recommended interventions face barriers to real- world implementation. Importantly, the core three-sphere assessment relies exclusively on validated, freely available clinical instruments (MMSE, GDS-15, ADL, IADL, SPPB) that are accessible in primary and outpatient care worldwide, including low- and middle-income settings; biomarker-based staging tools are presented as supplementary aids where available, not as prerequisites for applying the framework. As a narrative review organized around a conceptual framework, this paper carries an inherent risk of evidence selection bias: studies emphasizing factors outside the three-sphere model—including social determinants of health, environmental exposures, early-life risk factors, and structural healthcare inequities—may be underrepresented. Readers are encouraged to complement this framework with systematic reviews of specific management questions on implementation including cost, access, and workforce limitations, and with epidemiological literature on dementia risk that extends beyond the clinical encounter. Clinical judgment remains essential and cannot be replaced by any framework.

## 10. Future Directions

Several lines of future development emerge from this perspective. One is the refinement of biomarker-supported trajectory mapping: understanding not only whether a patient carries amyloid, tau, synuclein, or vascular burden, but how those biological signals relate to the order of sphere involvement and to the pace of multidomain progression. A second is the systematic integration of artificial intelligence and digital phenotyping tools into sphere-aware clinical practice. AI-enabled approaches are already reshaping specific aspects of geriatric neurology beyond future projection. In the cognitive sphere, natural language processing algorithms applied to clinical notes and speech recordings can detect early semantic and syntactic changes predictive of conversion to dementia with sensitivity exceeding standard screening instruments. In the motor sphere, wearable accelerometers and machine-learning-based gait analysis platforms can identify sphere-relevant deterioration—reduced gait speed, increased step variability, nocturnal motor activity changes—continuously and passively, without requiring clinic attendance. In the behavioural sphere, ambient sensor systems and caregiver-facing applications are being validated for automated BPSD detection in home environments. The paradigm shift lies in multimodal data fusion: AI models trained on simultaneous inputs from imaging biomarkers, wearable physiology, electronic health record trajectories, and genomic data are beginning to produce individual-level prognostic predictions that no single domain tool can replicate. The three-sphere framework provides the clinical interpretive layer that such models will require to translate outputs into actionable care decisions—a complementarity rather than a competition between structured clinical classification and machine-generated phenotyping. A third line of development is the construction of trajectory-sensitive clinical trials that include not only cognitive endpoints but behavioural, functional, gait-related, sleep-related, and caregiver-centred outcomes, and that disaggregate results by frailty status and comorbidity burden. Finally, there is a pressing need for stronger evidence on deprescribing, integrated care, and palliative transitions in neurodegenerative disease, because these are among the most decisive determinants of quality of care in the oldest and most complex patients [7,12,15,20,23,25,26,33,34].

## 11. Conclusions

Neurodegenerative diseases in older adults should be interpreted as evolving clinical trajectories rather than as fixed diagnostic entities alone. Alzheimer’s disease, frontotemporal dementia, Parkinsonian syndromes, dementia with Lewy bodies, and vascular cognitive impairment each display a different pattern of entry into the clinical arena, but all ultimately challenge the clinician through the interaction of cognition, behaviour, and motor function across time. Understanding which sphere is dominant, which is emerging, and which is being worsened by treatment is often more useful for real-world care than relying on diagnosis alone [4,8,9,10,11,12,15].

The three-sphere framework proposed in this review offers a practical and conceptually coherent model for precision geriatric neurology. It helps clinicians connect biological heterogeneity to symptom evolution, anticipate therapeutic trade-offs, reduce prescription cascade, recognize the disruptive role of delirium, and integrate pharmacological and non-pharmacological care more rationally. It also supports earlier advance care planning and more realistic transitions toward palliative goals when the balance of benefit and burden changes. Ultimately, this trajectory-based approach brings medical interpretation closer to the lived reality of older adults with neurodegeneration and offers a more useful framework for research, therapeutic judgment, and person-centred care across the full course of disease [16,17,23,26,27,33,34,35]. The practical implications of this framework are threefold. For clinicians, it suggests that every therapeutic decision in older adults with neurodegeneration should be preceded by an explicit identification of the currently dominant sphere, an anticipation of cross-sphere trade-offs, and a reassessment of whether treatment goals remain consistent with disease stage. For researchers, it argues for the systematic inclusion of behavioural, functional, and motor endpoints alongside cognitive outcomes in future trials, and for the disaggregation of results by frailty status and comorbidity burden. For guideline developers, it highlights the need for disease-specific sections explicitly addressing polypharmacy risk, deprescribing triggers, and the transition criteria from disease-oriented to comfort-oriented care—elements that remain conspicuously underdeveloped in current neurology and geriatrics guidelines.

## Data Availability

No new data were created or analysed in this study.

## References

[1] GBD 2019 Ageing Collaborators (2022). Global, regional, and national burden of diseases and injuries for adults 70 years and older: Systematic analysis for the Global Burden of Disease 2019 Study. BMJ.

[2] (2018). The US Burden of Disease Collaborators. The State of US Health, 1990–2016: Burden of Diseases, Injuries, and Risk Factors Among US States. JAMA.

[3] Nicholson K., Liu W., Fitzpatrick D., Hardacre K.A., Roberts S., Salerno J., Stranges S., Fortin M., Mangin D. (2024). Prevalence of multimorbidity and polypharmacy among adults and older adults: A systematic review. Lancet Healthy Longev..

[4] Frisoni G.B., Hansson O., Nichols E., Garibotto V., Schindler S.E., van der Flier W.M., Jessen F., Villain N., Arenaza-Urquijo E.M., Crivelli L. (2025). New landscape of the diagnosis of Alzheimer’s disease. Lancet.

[5] Lauretani F., Testa C., Salvi M., Zucchini I., Lorenzi B., Tagliaferri S., Cattabiani C., Maggio M. (2022). Reward System Dysfunction and the Motoric-Cognitive Risk Syndrome in Older Persons. Biomedicines.

[6] Salvi M., Lauretani F., Brusco C.M., Maggio M. (2025). Sleep disorders across the parkinsonism continuum: A typical example of geriatric complexity. Neurodegener. Dis. Manag..

[7] Lauretani F., Maggio M., Pilotto A.M., Ansaldo M., Brusco C.M., Carbonaro M., Amendola C., Nabacino M., Testa C., Ciuni A. (2025). The Trajectories of Neuromuscular Aging (TRAJECTOR-AGE Clinical Trial): Study Rationale and Methodological Protocol. J. Am. Geriatr. Soc..

[8] Boeve B.F., Boxer A.L., Kumfor F., Pijnenburg Y., Rohrer J.D. (2022). Advances and controversies in frontotemporal dementia: Diagnosis, biomarkers, and therapeutic considerations. Lancet Neurol..

[9] Tanner C.M., Ostrem J.L. (2024). Parkinson’s Disease. N. Engl. J. Med..

[10] Armstrong M.J., Okun M.S. (2020). Diagnosis and Treatment of Parkinson Disease: A Review. JAMA.

[11] Kaila L.V., Savica R., Taylor J.-P., Chouliaras L., O’Brien J.T., Taylor J.-P. (2025). The evolving therapeutic landscape of dementia with Lewy bodies. Lancet Neurol..

[12] Zarkali A., Bartl M., Fox N.C., Tan L.C.S., Mollenhauer B., Weil R.S. (2025). Diagnostic and other biomarkers of dementia with Lewy bodies: From research to clinical settings. Lancet Neurol..

[13] Wang S., Li Q., Wang S., Huang C., Xue Q.L., Szanton S.L., Liu M. (2024). Sustained frailty remission and dementia risk in older adults: A longitudinal study. Alzheimers Dement..

[14] Matison A.P., Samtani S., Brodaty H., Sachdev P.S., Reppermund S. (2025). How well do social frailty indices predict incident dementia in older adults?. J. Gerontol. B Psychol. Sci. Soc. Sci..

[15] Smith E.E., Aparicio H.J., Gottesman R.F., Goyal M.S., Greenberg S.M., Schneider J.A., Sorond F.A., Wright C.B., American Heart Association Stroke Council, Council on Cardiovascular and Stroke Nursing (2025). Vascular Contributions to Cognitive Impairment and Dementia in the United States: Prevalence and Incidence: A Scientific Statement from the American Heart Association. Stroke.

[16] Weidmann A.E., Proppé G.B., Matthíasdóttir R., Tadić I., Gunnarsson P.S., Jónsdóttir F. (2025). Medication-induced causes of delirium in patients with and without dementia: A systematic review of published neurology guidelines. Int. J. Clin. Pharm..

[17] Weidmann A.E., Matthíasdóttir R., Proppé G.B., Tadić I., Gunnarsson P.S., Jónsdóttir F. (2025). Medication Causes and Treatment of Delirium in Patients With and Without Dementia. Brain Behav..

[18] Jack C.R., Andrews J.S., Beach T.G., Buracchio T., Dunn B., Graf A., Hansson O., Ho C., Jagust W., McDade E. (2024). Revised criteria for diagnosis and staging of Alzheimer’s disease: Alzheimer’s Association Workgroup. Alzheimers Dement..

[19] Beach T.G., Monsell S.E., Phillips L.E., Kukull W. (2012). Accuracy of the clinical diagnosis of Alzheimer disease at National Institute on Aging Alzheimer’s Disease Centers, 2005–2010. J. Neuropathol. Exp. Neurol..

[20] Trudel L., Therriault J., Macedo A.C., Hosseini S.A., Fernandez-Arias J., Chan T., Rahmouni N., Bezgin G., Tissot C., Woo M.S. (2026). Clinical-biological Alzheimer’s disease stage concordance: Insights from cohorts and autopsy data. Brain.

[21] van Dyck C.H., Swanson C.J., Aisen P., Bateman R.J., Chen C., Gee M., Kanekiyo M., Li D., Reyderman L., Cohen S. (2023). Lecanemab in Early Alzheimer’s Disease. N. Engl. J. Med..

[22] Sims J.R., Zimmer J.A., Evans C.D., Lu M., Ardayfio P., Sparks J., Wessels A.M., Shcherbinin S., Wang H., Nery E.S.M. (2023). Donanemab in Early Symptomatic Alzheimer Disease: The TRAILBLAZER-ALZ 2 Randomized Clinical Trial. JAMA.

[23] Singh S., Metzger M., Tjia J., Antonelli M.T., Avula R., Crawford S.L., Dashevsky I., Fouayzi H., Harkins T.P., Mazor K.M. (2024). High-Risk Medications in Persons Living with Dementia: A Randomized Clinical Trial. JAMA Intern. Med..

[24] Nelson P.T., Brayne C., Flanagan M.E., Abner E.L., Agrawal S., Attems J., Castellani R.J., Corrada M.M., Cykowski M.D., Di J. (2022). Frequency of LATE neuropathologic change across the spectrum of Alzheimer’s disease neuropathology: Combined data from 13 community-based or population-based autopsy cohorts. Acta Neuropathol..

[25] Ghouri R., Öksüz N., Taşdelen B., Özge A. (2023). Factors affecting progression of non-Alzheimer dementia: A retrospective analysis with long-term follow-up. Front. Neurol..

[26] Kluger B.M., Hudson P., Hanson L.C., Bužgovàf R., Creutzfeldt C.J., Gursahani R., Sumrall M., Whitea C., Oliver D.J., Pantilat S.Z. (2023). Palliative care to support the needs of adults with neurological disease. Lancet Neurol..

[27] Mitchell S.L. (2015). Advanced Dementia. N. Engl. J. Med..

[28] Gromek K.R., Thorpe C.T., Aspinall S.L., Hanson L.C., Niznik J.D. (2023). Anticholinergic co-prescribing in nursing home residents using cholinesterase inhibitors: Potential deprescribing cascade. J. Am. Geriatr. Soc..

[29] Singh S., Cocoros N.M., Haynes K., Nair V.P., Harkins T.P., Rochon P.A., Platt R., Dashevsky I., Reynolds J., Mazor K.M. (2021). Antidopaminergic-antiparkinsonian medication prescribing cascade in persons with Alzheimer’s disease. J. Am. Geriatr. Soc..

[30] Trenaman S.C., Bowles S.K., Kirkland S., Andrew M.K. (2021). An examination of three prescribing cascades in a cohort of older adults with dementia. BMC Geriatr..

[31] Cho E., Kong E.H. (2024). Symptom-specific non-pharmacological interventions for behavioral and psychological symptoms of dementia: An umbrella review. Int. J. Nurs. Stud..

[32] Kong Q., Huang K., Li S., Li X., Han R., Yang H., Pu Y., Chen L., Jia Y. (2025). Effectiveness of various exercise on neuropsychiatric symptoms among older adults with mild cognitive impairment or dementia: A systematic review and network meta-analysis. Ageing Res. Rev..

[33] Deardorff W.J., Barnes D.E., Jeon S.Y., Boscardin W.J., Langa K.M., Covinsky K.E., Mitchell S.L., Whitlock E.L., Smith A.K., Lee S.J. (2022). Development and External Validation of a Mortality Prediction Model for Community-Dwelling Older Adults With Dementia. JAMA Intern. Med..

[34] Chang Y.K., Philip J., van der Steen J.T., Block L.V.D., Hum A.Y.M., Pérez-Cruz P.E., Paiva C., Mori M., Chen P.-J., Agar M.R. (2025). Referral Criteria for Specialist Palliative Care for Patients with Dementia. JAMA Netw. Open.

[35] American Geriatrics Society Ethics Committee and Clinical Practice and Models of Care Committee (2014). American Geriatrics Society feeding tubes in advanced dementia position statement. J. Am. Geriatr. Soc..

